# Supplementary material for: Intracellular complement Factor B couples inflammatory sensing to metabolism, chromatin regulation and maladaptive tissue repair
Source: bioRxiv. 2026 Oct 11:2026.10.04.756581. Preprint. [Version 1] doi: 10.64898/2026.10.04.756581 (PMC13692131; doi:10.64898/2026.10.04.756581)
Supplement: Supplementary file 1 [file media-1.pdf]

Figure S1

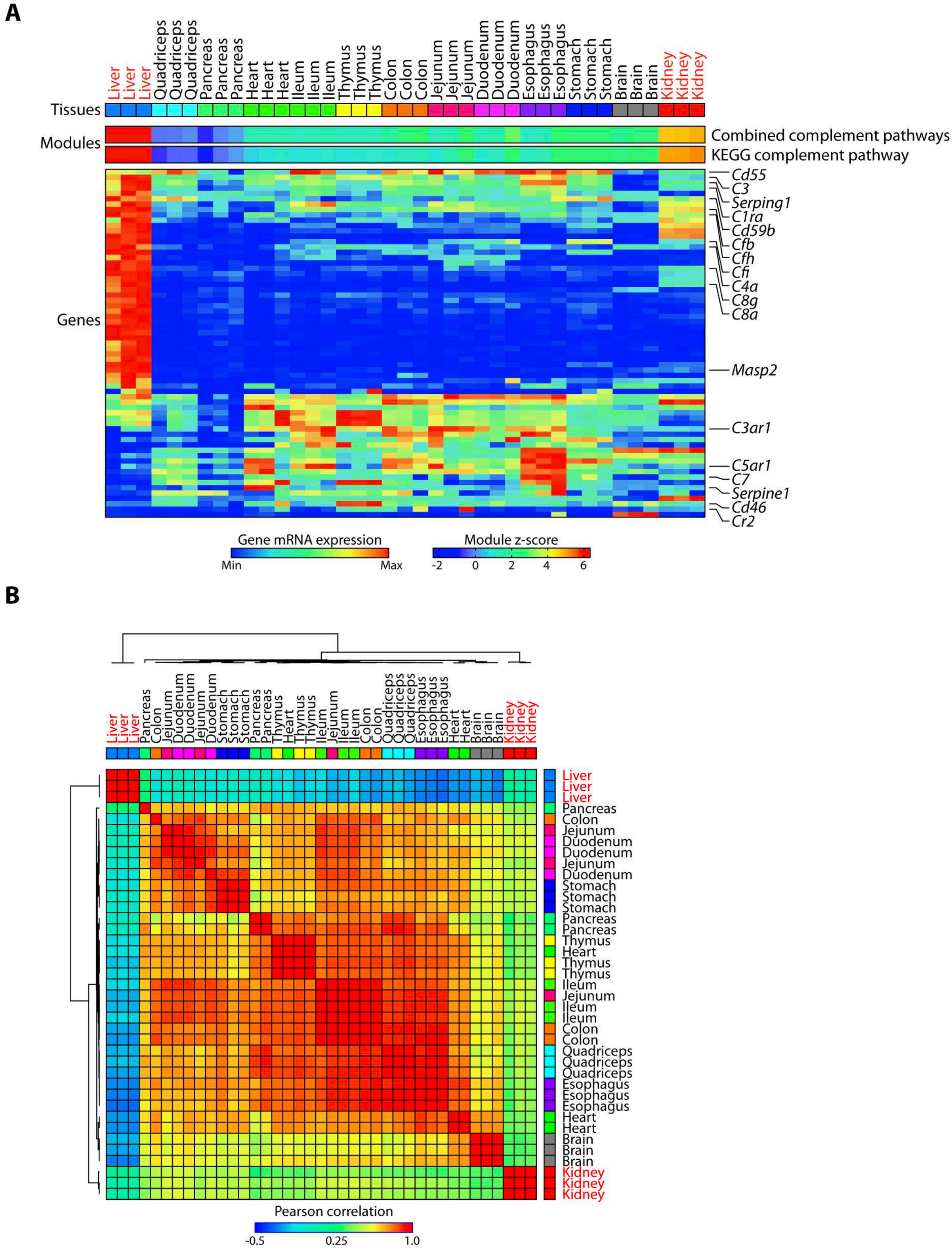

A
Liver
Liver
Liver
Quadriceps
Quadriceps
Quadriceps
Pancreas
Pancreas
Pancreas
Heart
Heart
Heart
Ileum
Ileum
Ileum
Thymus
Thymus
Thymus
Colon
Colon
Colon
Jejunum
Jejunum
Jejunum
Duodenum
Duodenum
Duodenum
Esophagus
Esophagus
Esophagus
Stomach
Stomach
Stomach
Brain
Brain
Brain
Kidney
Kidney
Kidney
Tissues
Modules
Combined complement pathways
KEGG complement pathway
Genes
Cd55
C3
Serping1
C1ra
Cd59b
Cfb
Cfh
Cfi
C4a
C8g
C8a
Masp2
C3ar1
C5ar1
C7
Serpine1
Cd46
Cr2
Gene mRNA expression
Min
Max
Module z-score
-2
0
2
4
6
B
Pearson correlation
-0.5
0.25
1.0

Figure S2

A

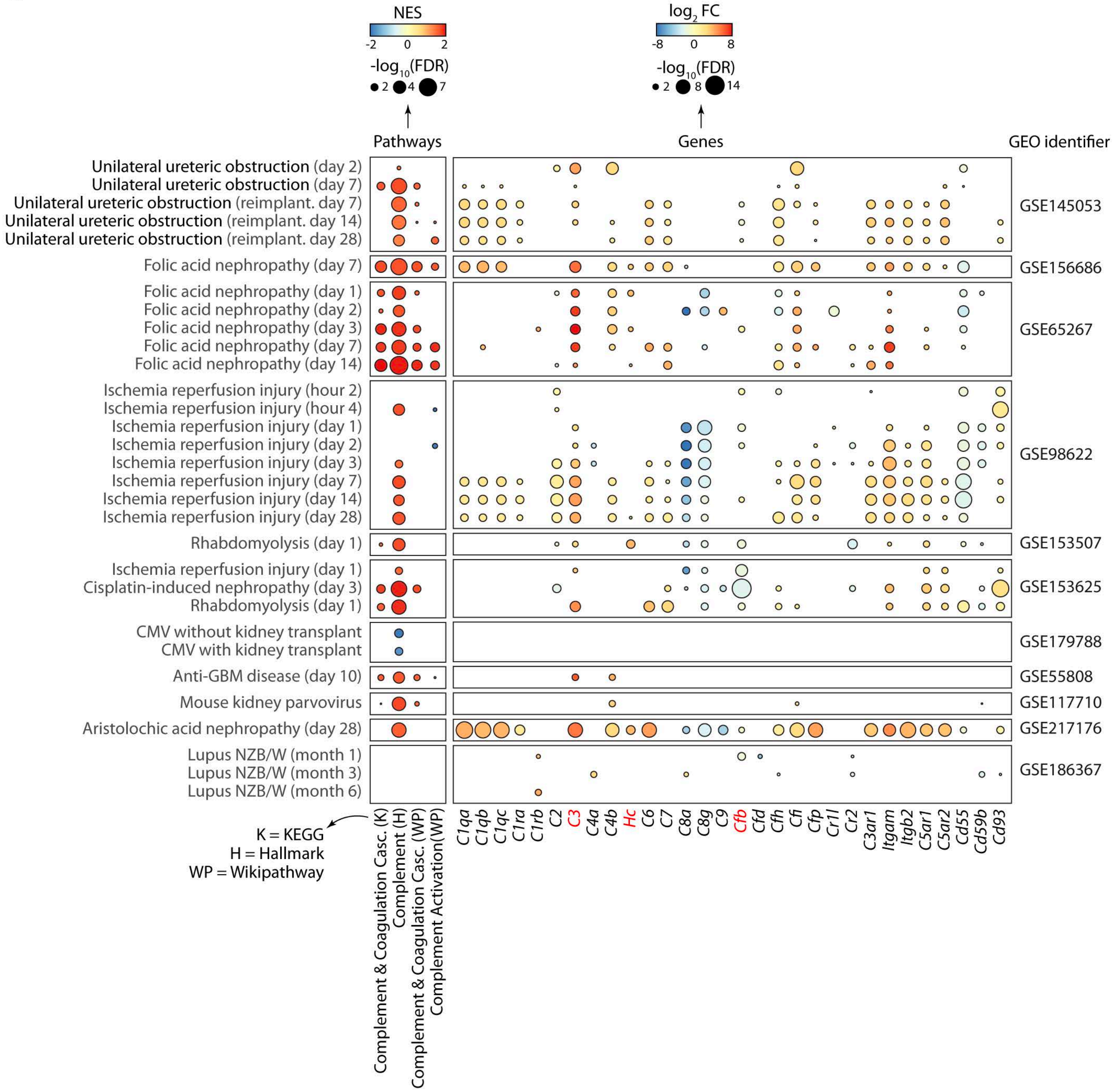

NES
-2 0 2
$-\log_{10}$(FDR)
2 4 7
$\log_2$ FC
-8 0 8
$-\log_{10}$(FDR)
2 8 14
Pathways
Genes
GEO identifier
Unilateral ureteric obstruction (day 2)
Unilateral ureteric obstruction (day 7)
Unilateral ureteric obstruction (reimplant. day 7)
Unilateral ureteric obstruction (reimplant. day 14)
Unilateral ureteric obstruction (reimplant. day 28)
GSE145053
Folic acid nephropathy (day 7)
GSE156686
Folic acid nephropathy (day 1)
Folic acid nephropathy (day 2)
Folic acid nephropathy (day 3)
Folic acid nephropathy (day 7)
Folic acid nephropathy (day 14)
GSE65267
Ischemia reperfusion injury (hour 2)
Ischemia reperfusion injury (hour 4)
Ischemia reperfusion injury (day 1)
Ischemia reperfusion injury (day 2)
Ischemia reperfusion injury (day 3)
Ischemia reperfusion injury (day 7)
Ischemia reperfusion injury (day 14)
Ischemia reperfusion injury (day 28)
GSE98622
Rhabdomyolysis (day 1)
GSE153507
Ischemia reperfusion injury (day 1)
Cisplatin-induced nephropathy (day 3)
Rhabdomyolysis (day 1)
GSE153625
CMV without kidney transplant
CMV with kidney transplant
GSE179788
Anti-GBM disease (day 10)
GSE55808
Mouse kidney parvovirus
GSE117710
Aristolochic acid nephropathy (day 28)
GSE217176
Lupus NZB/W (month 1)
Lupus NZB/W (month 3)
Lupus NZB/W (month 6)
GSE186367
K = KEGG
H = Hallmark
WP = Wikipathway
Complement & Coagulation Casc. (K)
Complement (H)
Complement & Coagulation Casc. (WP)
Complement Activation(WP)
C1qa
C1qb
C1qc
C1ra
C1rb
C2
C3
C4a
C4b
Hc
C6
C7
C8a
C8g
C9
Cfb
Cfd
Cfh
Cfi
Cfp
Cr1l
Cr2
C3ar1
Itgam
Itgb2
C5ar1
C5ar2
Cd55
Cd59b
Cd93

Figure S3

A

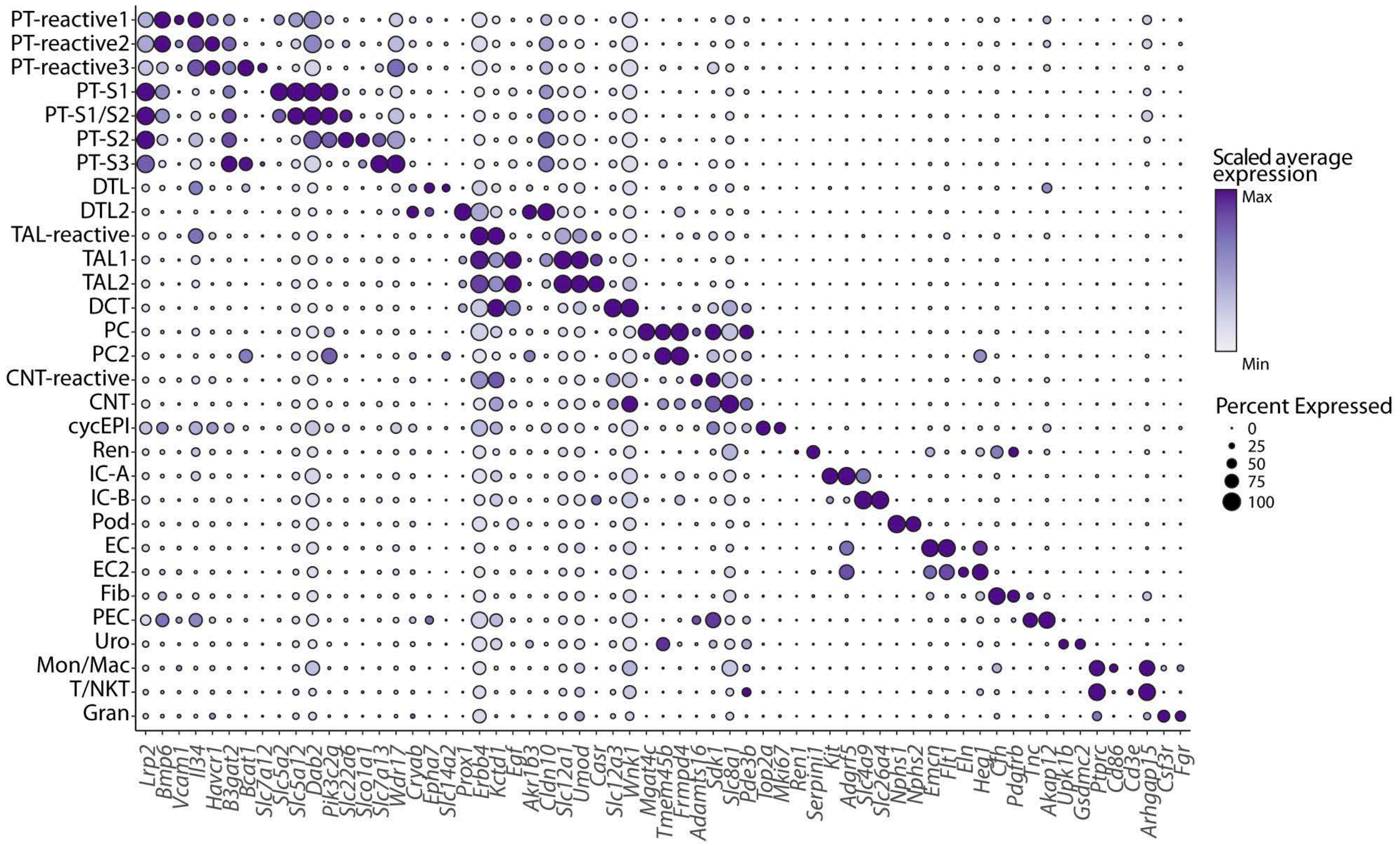

B

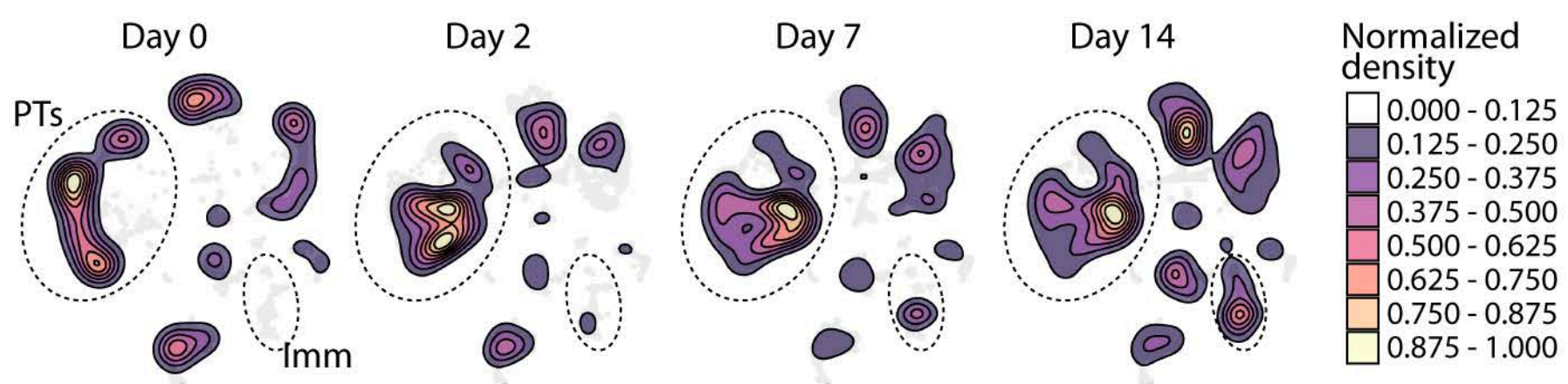

C

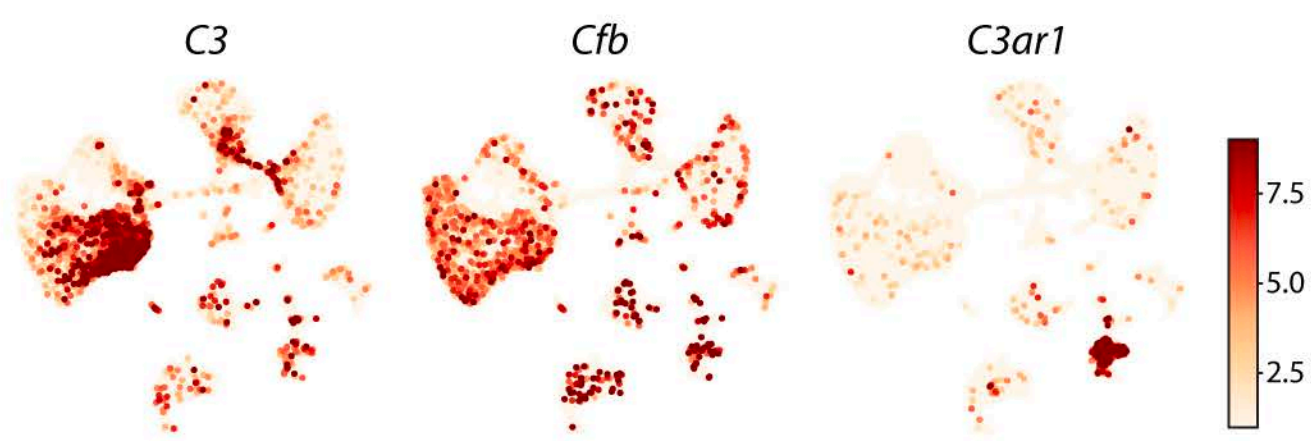

D

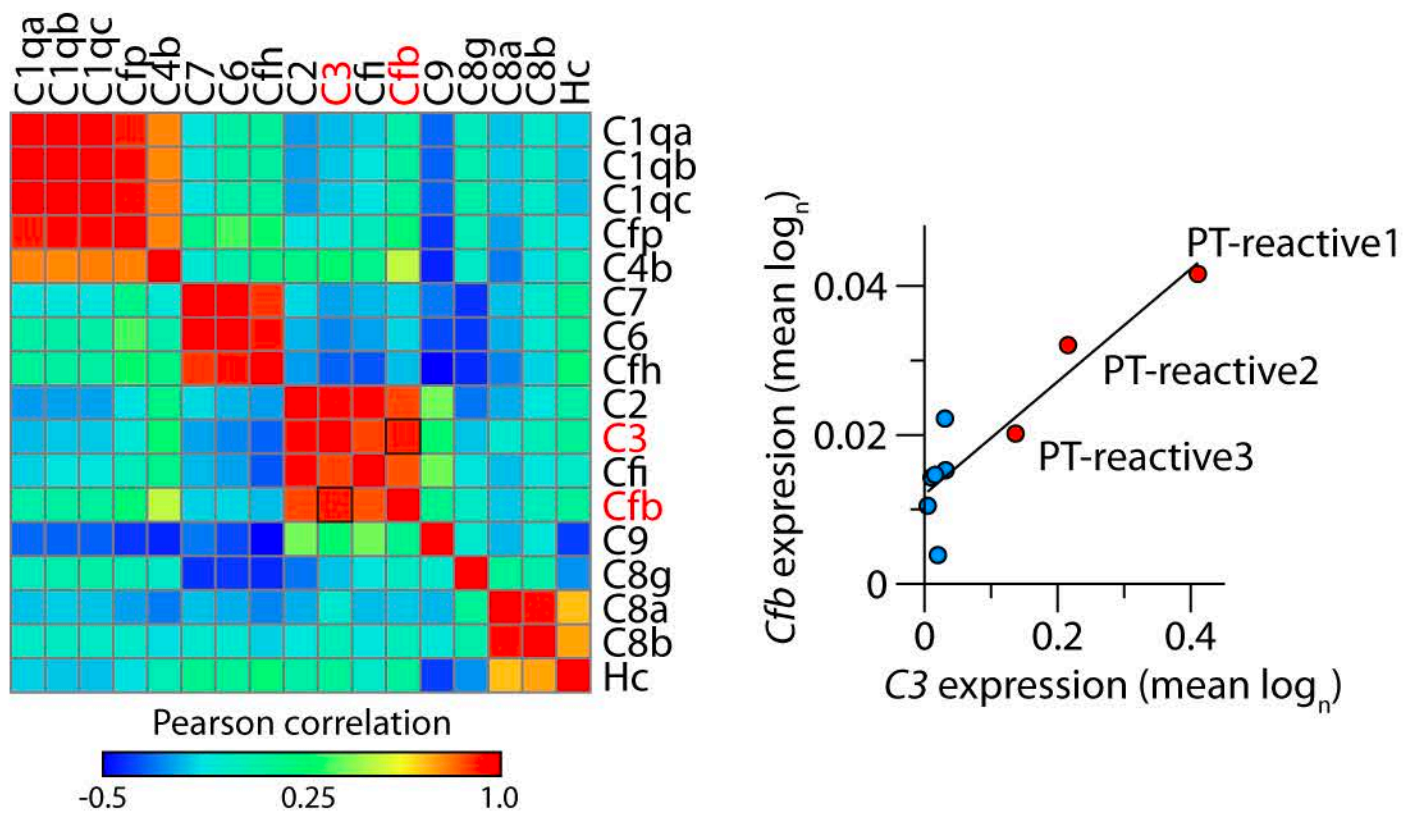

Figure S4

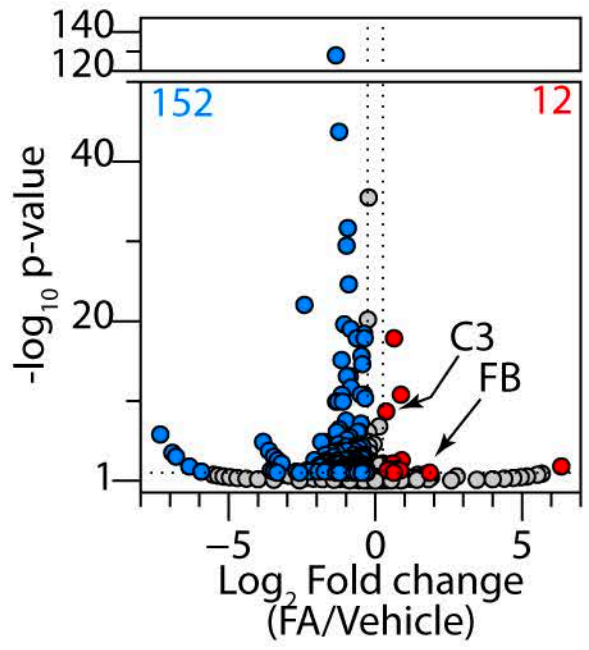

152
12
C3
FB
140
120
40
20
1
-log10 p-value
−5
0
5
Log2 Fold change
(FA/Vehicle)

Figure S5

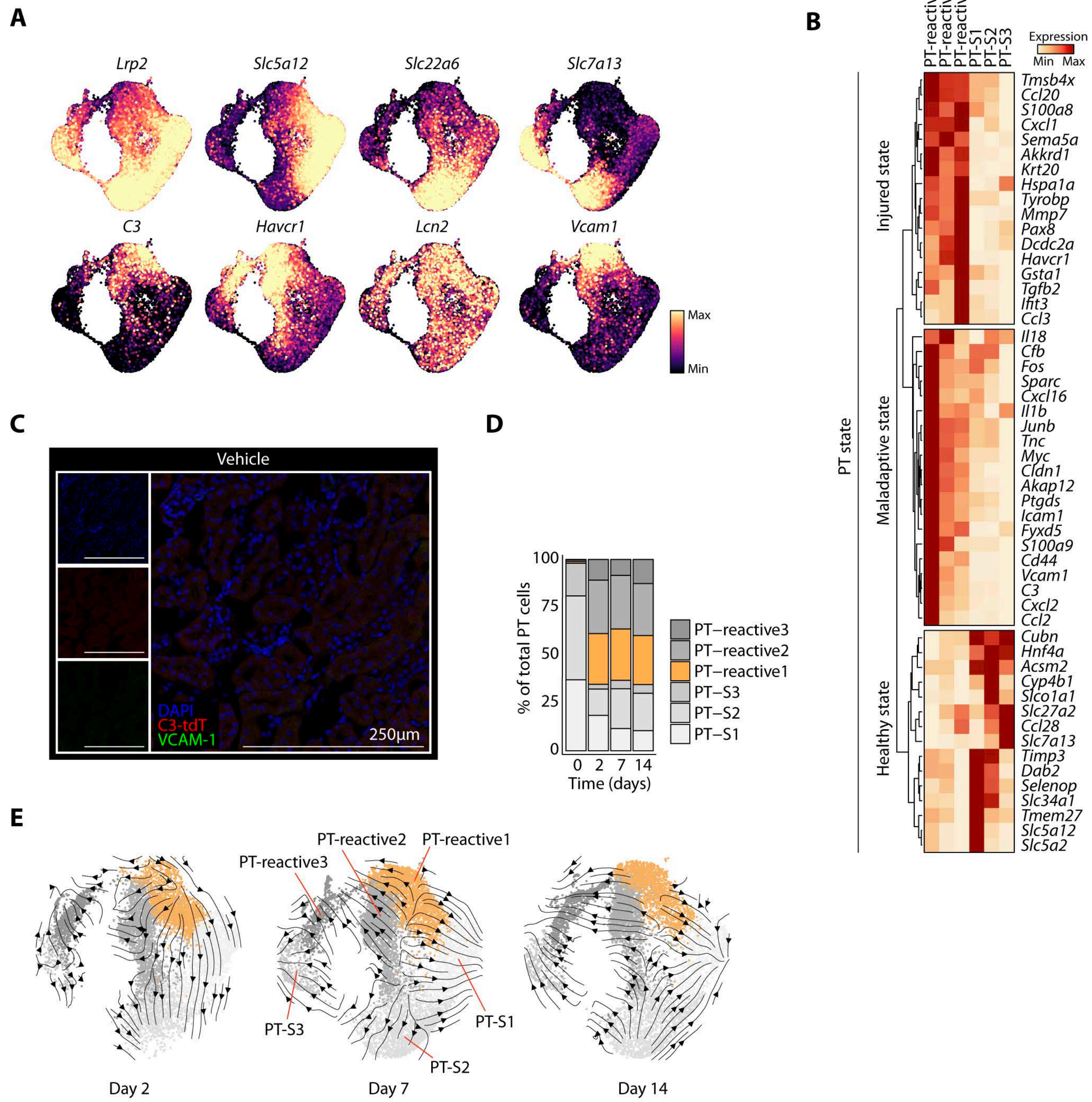
A
Lrp2
Slc5a12
Slc22a6
Slc7a13
C3
Havcr1
Lcn2
Vcam1
Max
Min
B
PT-reactive1
PT-reactive2
PT-reactive3
PT-S1
PT-S2
PT-S3
Expression
Min Max
PT state
Injured state
Maladaptive state
Healthy state
Tmsb4x
Ccl20
S100a8
Cxcl1
Sema5a
Akkrd1
Krt20
Hspa1a
Tyrobp
Mmp7
Pax8
Dcdc2a
Havcr1
Gsta1
Tgfb2
Ifit3
Ccl3
Il18
Cfb
Fos
Sparc
Cxcl16
Il1b
Junb
Tnc
Myc
Cldn1
Akap12
Ptgds
Icam1
Fyxd5
S100a9
Cd44
Vcam1
C3
Cxcl2
Ccl2
Cubn
Hnf4a
Acsm2
Cyp4b1
Slco1a1
Slc27a2
Ccl28
Slc7a13
Timp3
Dab2
Selenop
Slc34a1
Tmem27
Slc5a12
Slc5a2
C
Vehicle
DAPI
C3-tdT
VCAM-1
250μm
D
% of total PT cells
100
75
50
25
0
0 2 7 14
Time (days)
PT–reactive3
PT–reactive2
PT–reactive1
PT–S3
PT–S2
PT–S1
E
PT-reactive2
PT-reactive1
PT-reactive3
PT-S3
PT-S1
PT-S2
Day 2
Day 7
Day 14

Figure S6

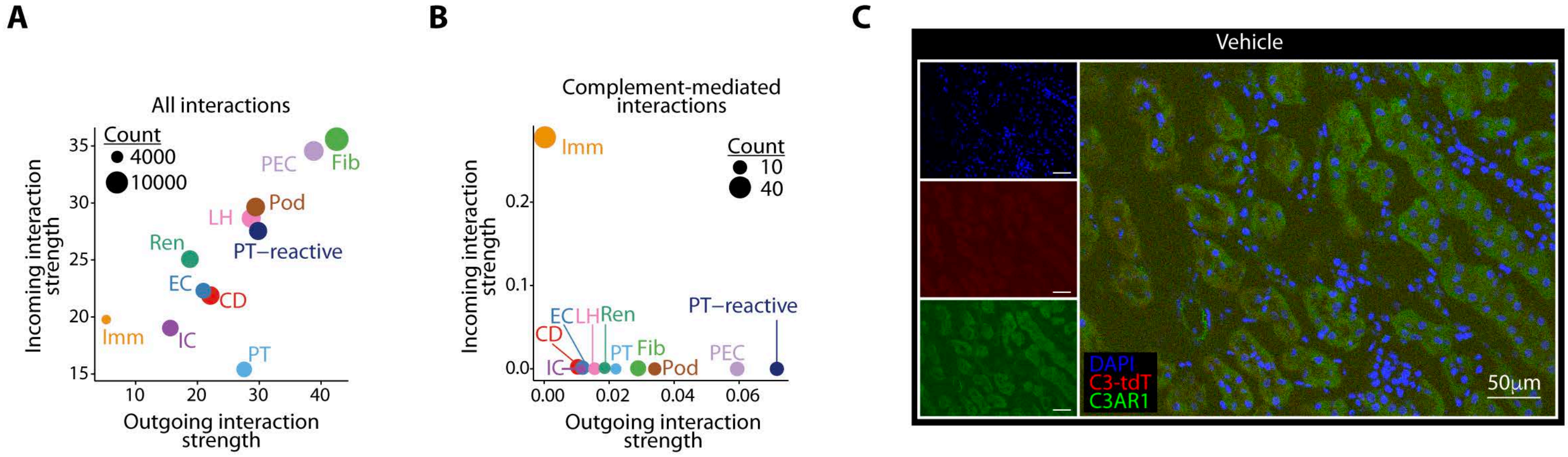
A
All interactions
Count
4000
10000
Incoming interaction strength
Outgoing interaction strength
PEC
Fib
Pod
LH
Ren
PT−reactive
EC
CD
Imm
IC
PT
B
Complement-mediated interactions
Imm
Count
10
40
Incoming interaction strength
Outgoing interaction strength
EC
LH
Ren
CD
PT−reactive
IC
PT
Fib
Pod
PEC
C
Vehicle
DAPI
C3-tdT
C3AR1
50μm

Figure S7

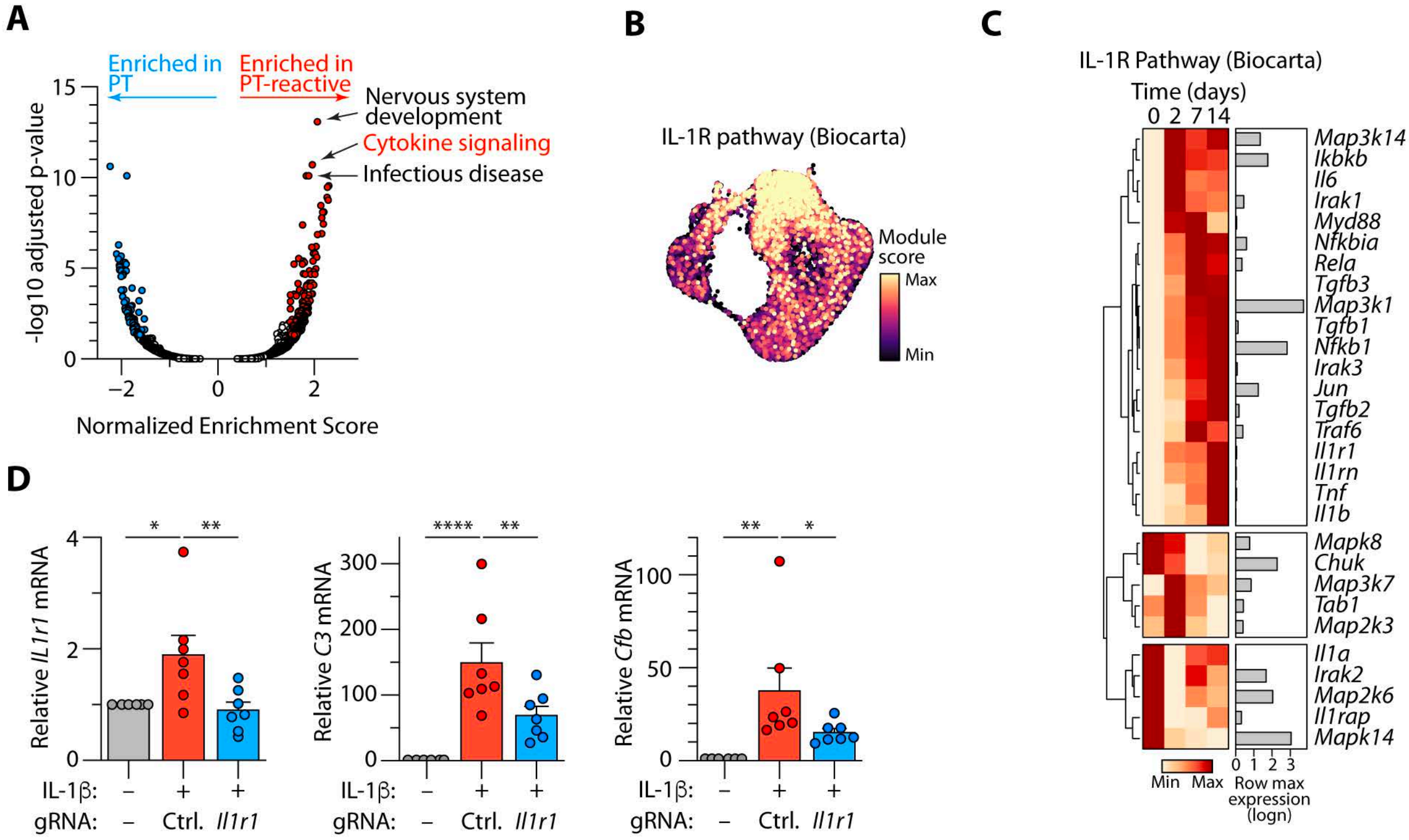
A
Enriched in PT
Enriched in PT-reactive
Nervous system development
Cytokine signaling
Infectious disease
-log10 adjusted p-value
Normalized Enrichment Score
B
IL-1R pathway (Biocarta)
Module score
Max
Min
C
IL-1R Pathway (Biocarta)
Time (days)
0 2 7 14
Map3k14
Ikbkb
Il6
Irak1
Myd88
Nfkbia
Rela
Tgfb3
Map3k1
Tgfb1
Nfkb1
Irak3
Jun
Tgfb2
Traf6
Il1r1
Il1rn
Tnf
Il1b
Mapk8
Chuk
Map3k7
Tab1
Map2k3
Il1a
Irak2
Map2k6
Il1rap
Mapk14
Min Max
Row max expression (logn)
D
Relative Il1r1 mRNA
Relative C3 mRNA
Relative Cfb mRNA
IL-1β:
gRNA:
Ctrl.
Il1r1

Figure S8

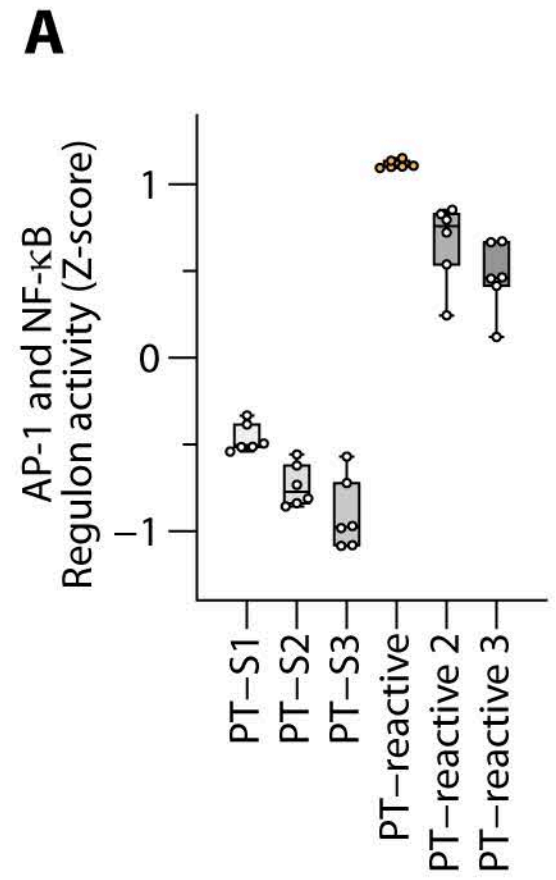
A
AP-1 and NF-κB
Regulon activity (Z-score)
1
0
−1
PT−S1
PT−S2
PT−S3
PT−reactive
PT−reactive 2
PT−reactive 3

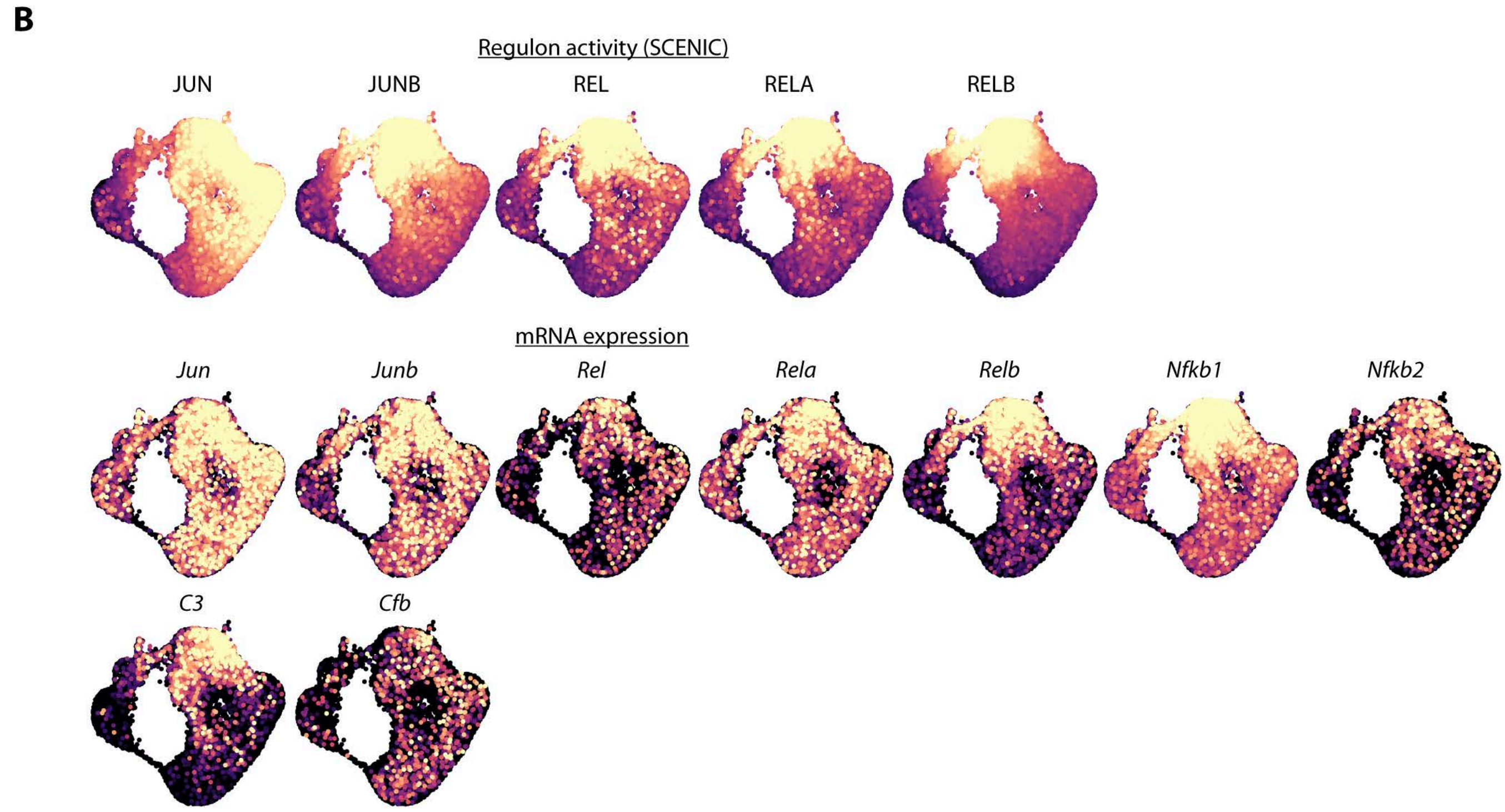
B
Regulon activity (SCENIC)
JUN
JUNB
REL
RELA
RELB
mRNA expression
Jun
Junb
Rel
Rela
Relb
Nfkb1
Nfkb2
C3
Cfb

Figure S9

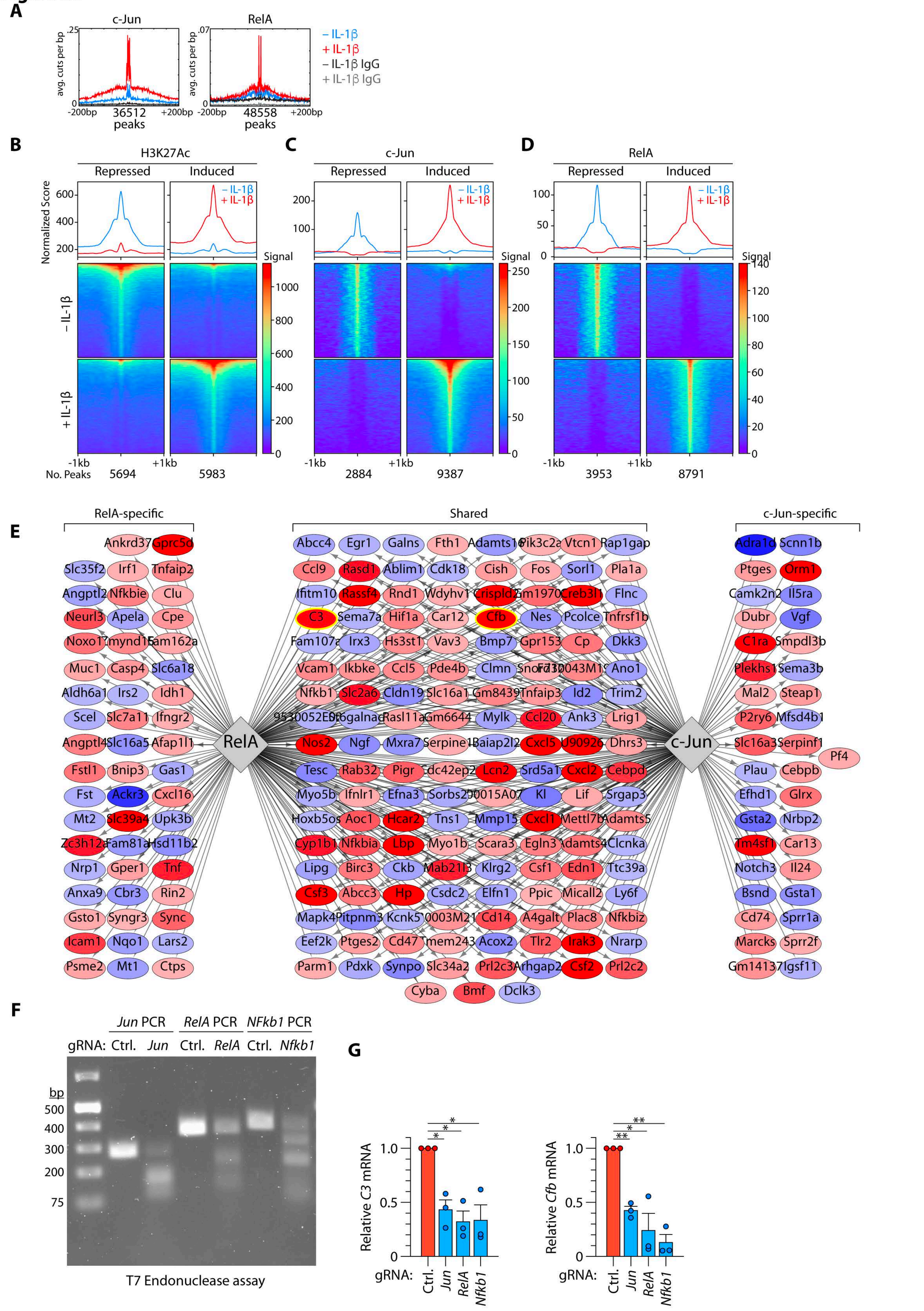
A
c-Jun
RelA
avg. cuts per bp
.25
.07
0
-200bp
+200bp
36512 peaks
48558 peaks
– IL-1β
+ IL-1β
– IL-1β IgG
+ IL-1β IgG
B
H3K27Ac
C
c-Jun
D
RelA
Repressed
Induced
Normalized Score
Signal
– IL-1β
+ IL-1β
-1kb
+1kb
No. Peaks
5694
5983
2884
9387
3953
8791
E
RelA-specific
Shared
c-Jun-specific
RelA
c-Jun
Ankrd37
Gprc5d
Slc35f2
Irf1
Tnfaip2
Angptl2
Nfkbie
Clu
Neurl3
Apela
Cpe
Muc1
Casp4
Slc6a18
Aldh6a1
Irs2
Idh1
Scel
Slc7a11
Ifngr2
Slc16a5
Afap1l1
Fstl1
Bnip3
Gas1
Fst
Ackr3
Cxcl16
Mt2
Slc39a4
Upk3b
Nrp1
Gper1
Tnf
Anxa9
Cbr3
Rin2
Gsto1
Syngr3
Sync
Icam1
Nqo1
Lars2
Psme2
Mt1
Ctps
Abcc4
Egr1
Galns
Fth1
Vtcn1
Rap1gap
Ccl9
Rasd1
Ablim1
Cdk18
Cish
Fos
Sorl1
Pla1a
Rassf4
Rnd1
Wdyhv1
Flnc
C3
Sema7a
Hif1a
Car12
Cfb
Nes
Pcolce
Tnfrsf1b
Irx3
Hs3st1
Vav3
Bmp7
Gpr153
Cp
Dkk3
Vcam1
Ikbke
Ccl5
Pde4b
Clmn
Ano1
Nfkb1
Slc2a6
Cldn19
Slc16a1
Id2
Trim2
Mylk
Ccl20
Ank3
Lrig1
Nos2
Ngf
Mxra7
Cxcl5
Dhrs3
Tesc
Rab32
Pigr
Lcn2
Srd5a1
Cxcl2
Cebpd
Myo5b
Ifnlr1
Efna3
Kl
Lif
Srgap3
Aoc1
Hcar2
Tns1
Mmp15
Cxcl1
Adamts5
Cyp1b1
Nfkbia
Lbp
Myo1b
Scara3
Egln3
Clcnka
Lipg
Birc3
Ckb
Klrg2
Csf1
Edn1
Ttc39a
Csf3
Abcc3
Hp
Csdc2
Elfn1
Ppic
Micall2
Ly6f
Mapk4
Kcnk5
Cd14
A4galt
Plac8
Nfkbiz
Eef2k
Ptges2
Cd47
Acox2
Tlr2
Irak3
Nrarp
Parm1
Pdxk
Synpo
Slc34a2
Prl2c3
Csf2
Prl2c2
Cyba
Bmf
Dclk3
Scnn1b
Ptges
Orm1
Camk2n2
Il5ra
Dubr
Vgf
C1ra
Smpdl3b
Plekhs1
Sema3b
Mal2
Steap1
P2ry6
Mfsd4b1
Slc16a3
Serpinf1
Pf4
Plau
Cebpb
Efhd1
Glrx
Gsta2
Nrbp2
Tm4sf1
Car13
Notch3
Il24
Bsnd
Gsta1
Cd74
Sprr1a
Marcks
Sprr2f
Gm14137
Igsf11
F
Jun PCR
RelA PCR
NFkb1 PCR
gRNA:
Ctrl.
Jun
RelA
Nfkb1
bp
500
400
300
200
75
T7 Endonuclease assay
G
Relative C3 mRNA
Relative Cfb mRNA
1.0
0.5
0
*
**
gRNA:
Ctrl.
Jun
RelA
Nfkb1

Figure S10

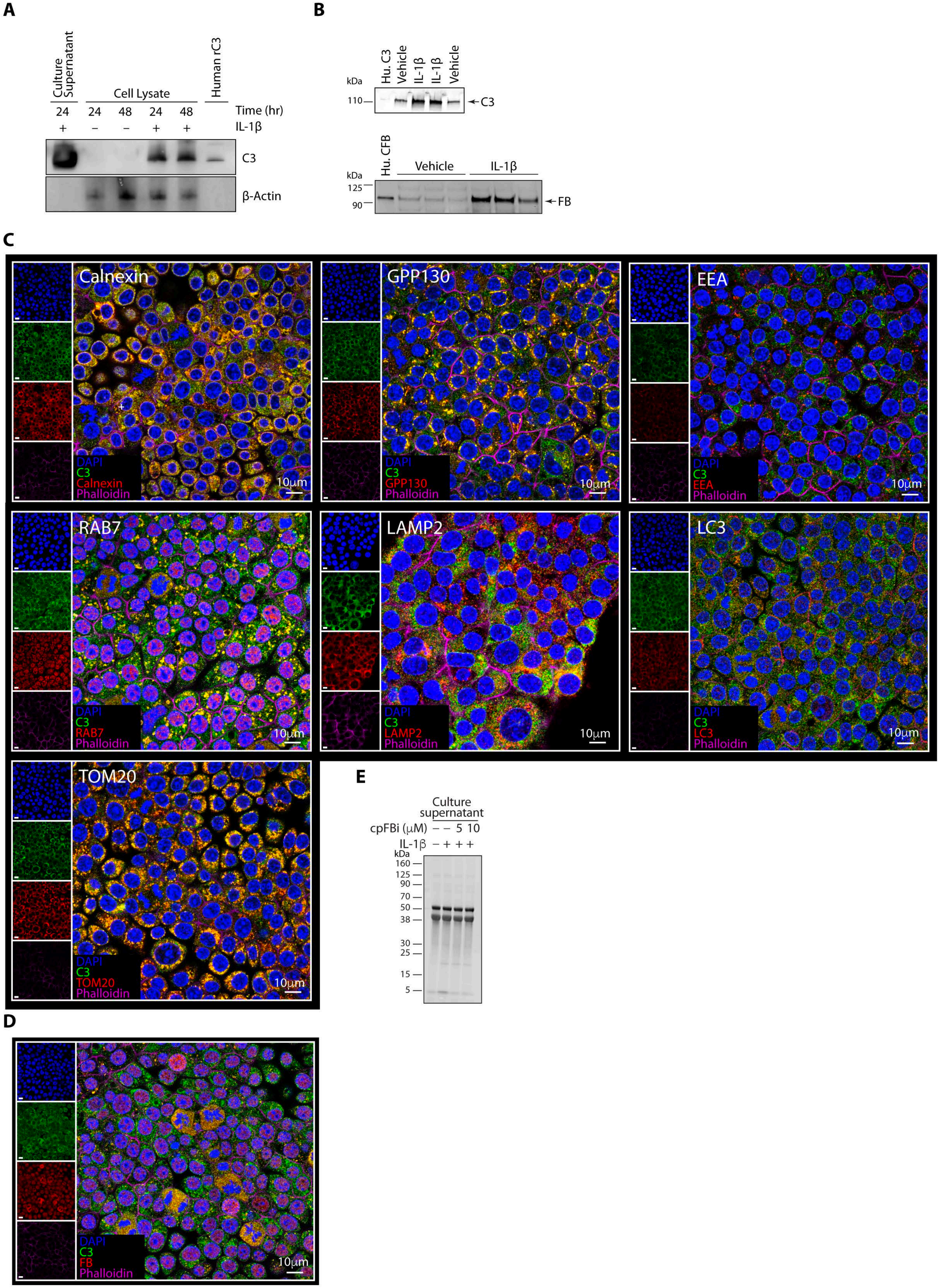
A
Culture Supernatant
Cell Lysate
Human rC3
24 24 48 24 48 Time (hr)
+ – – + + IL-1β
C3
β-Actin
B
kDa
Hu. C3
Vehicle
IL-1β
IL-1β
Vehicle
110
C3
Hu. CFB
Vehicle
IL-1β
125
90
FB
C
Calnexin
DAPI
C3
Calnexin
Phalloidin
10μm
GPP130
DAPI
C3
GPP130
Phalloidin
10μm
EEA
DAPI
C3
EEA
Phalloidin
10μm
RAB7
DAPI
C3
RAB7
Phalloidin
10μm
LAMP2
DAPI
C3
LAMP2
Phalloidin
10μm
LC3
DAPI
C3
LC3
Phalloidin
10μm
TOM20
DAPI
C3
TOM20
Phalloidin
10μm
D
DAPI
C3
FB
Phalloidin
10μm
E
Culture supernatant
cpFBi (μM) – – 5 10
IL-1β – + + +
kDa
160
125
90
70
50
38
30
25
15
5

Figure S11

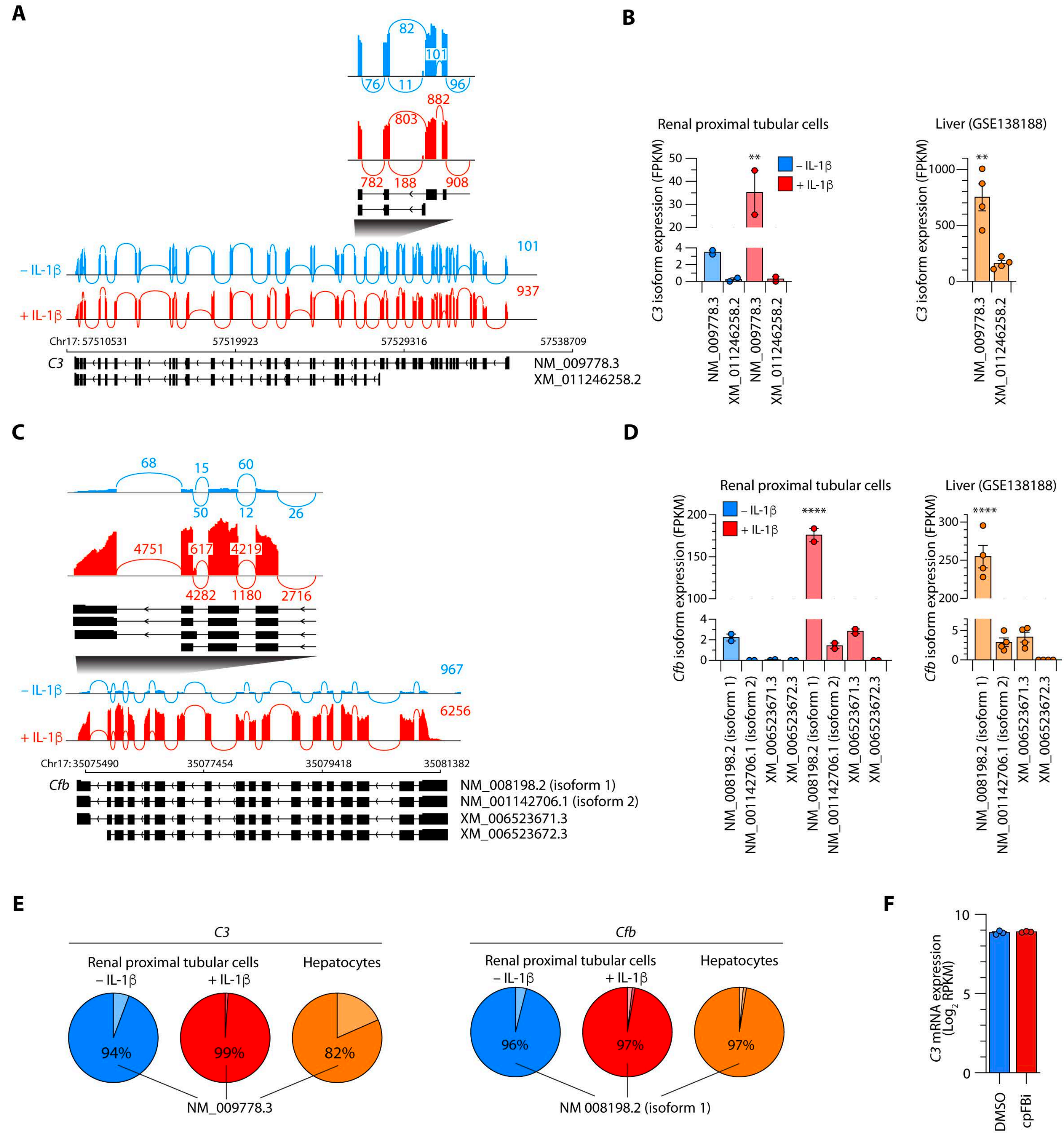

A
82
101
76
11
96
882
803
782
188
908
– IL-1β
+ IL-1β
101
937
Chr17: 57510531
57519923
57529316
57538709
C3
NM_009778.3
XM_011246258.2
B
Renal proximal tubular cells
Liver (GSE138188)
C3 isoform expression (FPKM)
– IL-1β
+ IL-1β
**
NM_009778.3
XM_011246258.2
C
68
15
60
50
12
26
4751
617
4219
4282
1180
2716
– IL-1β
+ IL-1β
967
6256
Chr17: 35075490
35077454
35079418
35081382
Cfb
NM_008198.2 (isoform 1)
NM_001142706.1 (isoform 2)
XM_006523671.3
XM_006523672.3
D
Renal proximal tubular cells
Liver (GSE138188)
Cfb isoform expression (FPKM)
****
E
C3
Renal proximal tubular cells
Hepatocytes
94%
99%
82%
NM_009778.3
Cfb
96%
97%
97%
NM 008198.2 (isoform 1)
F
C3 mRNA expression (Log2 RPKM)
DMSO
cpFBi

Figure S12

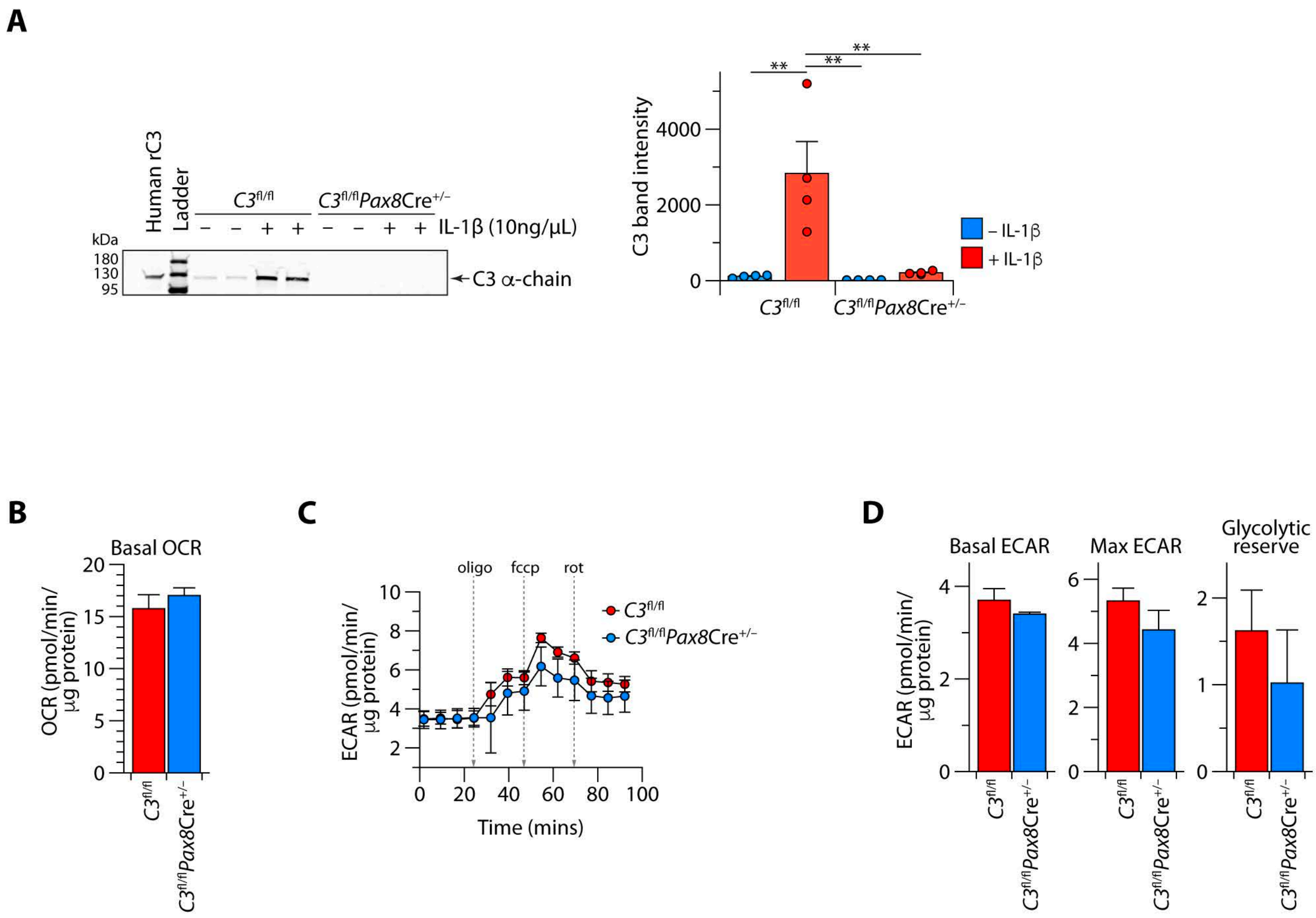

A
Human rC3
Ladder
C3fl/fl
C3fl/flPax8Cre+/−
IL-1β (10ng/μL)
kDa
180
130
95
C3 α-chain
C3 band intensity
4000
2000
0
**
**
**
– IL-1β
+ IL-1β
C3fl/fl
C3fl/flPax8Cre+/−
B
Basal OCR
OCR (pmol/min/ μg protein)
20
15
10
5
0
C3fl/fl
C3fl/flPax8Cre+/−
C
oligo
fccp
rot
ECAR (pmol/min/ μg protein)
10
8
6
4
2
0
20
40
60
80
100
Time (mins)
C3fl/fl
C3fl/flPax8Cre+/−
D
Basal ECAR
Max ECAR
Glycolytic reserve
ECAR (pmol/min/ μg protein)
4
2
0
6
4
2
0
2
1
0
C3fl/fl
C3fl/flPax8Cre+/−

Figure S13

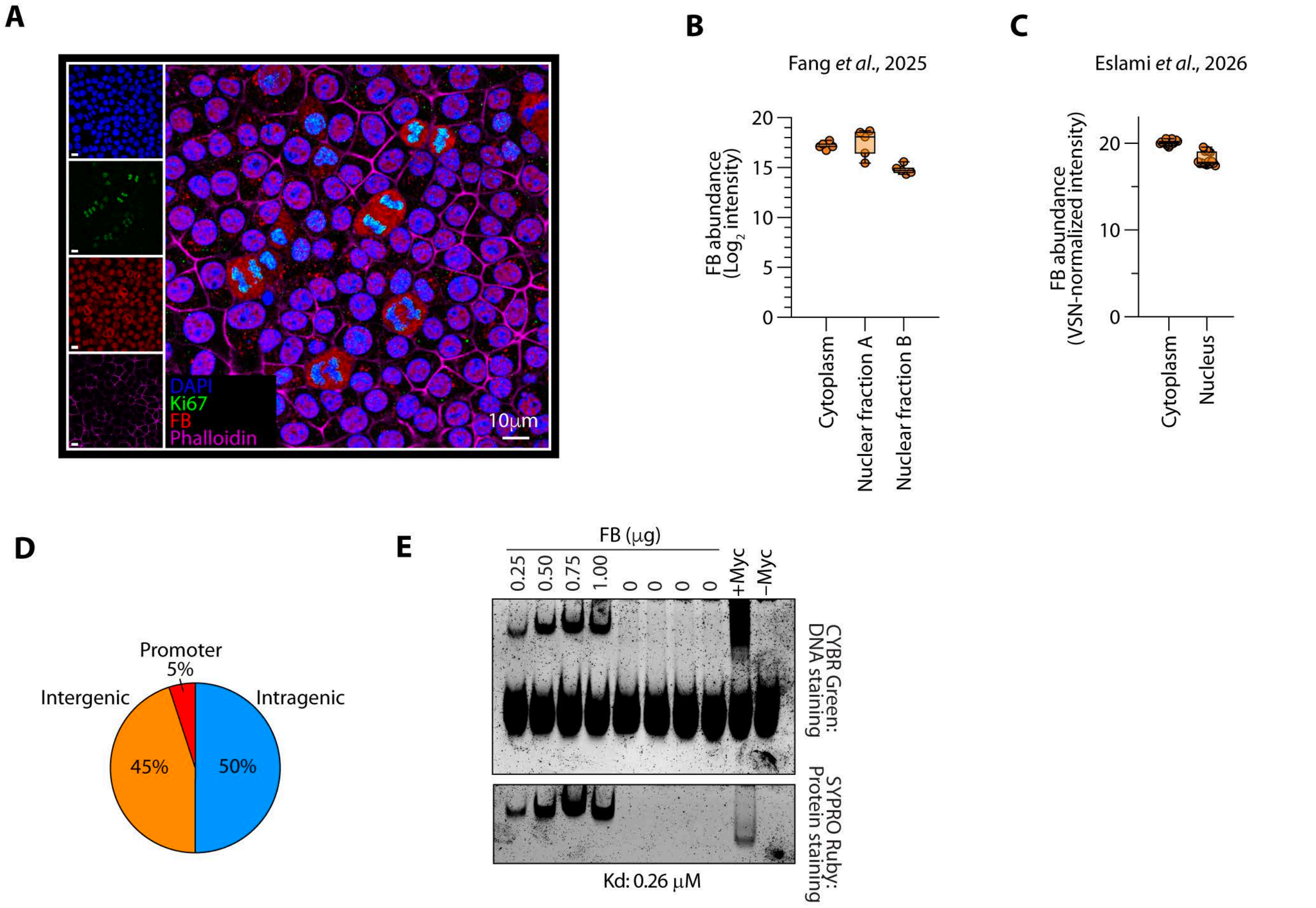
A
DAPI
Ki67
FB
Phalloidin
10μm
B
Fang et al., 2025
FB abundance (Log2 intensity)
0
5
10
15
20
Cytoplasm
Nuclear fraction A
Nuclear fraction B
C
Eslami et al., 2026
FB abundance (VSN-normalized intensity)
0
10
20
Cytoplasm
Nucleus
D
Promoter
5%
Intergenic
45%
Intragenic
50%
E
FB (μg)
0.25
0.50
0.75
1.00
0
0
0
0
+Myc
−Myc
CYBR Green: DNA staining
SYPRO Ruby: Protein staining
Kd: 0.26 μM

Figure S14

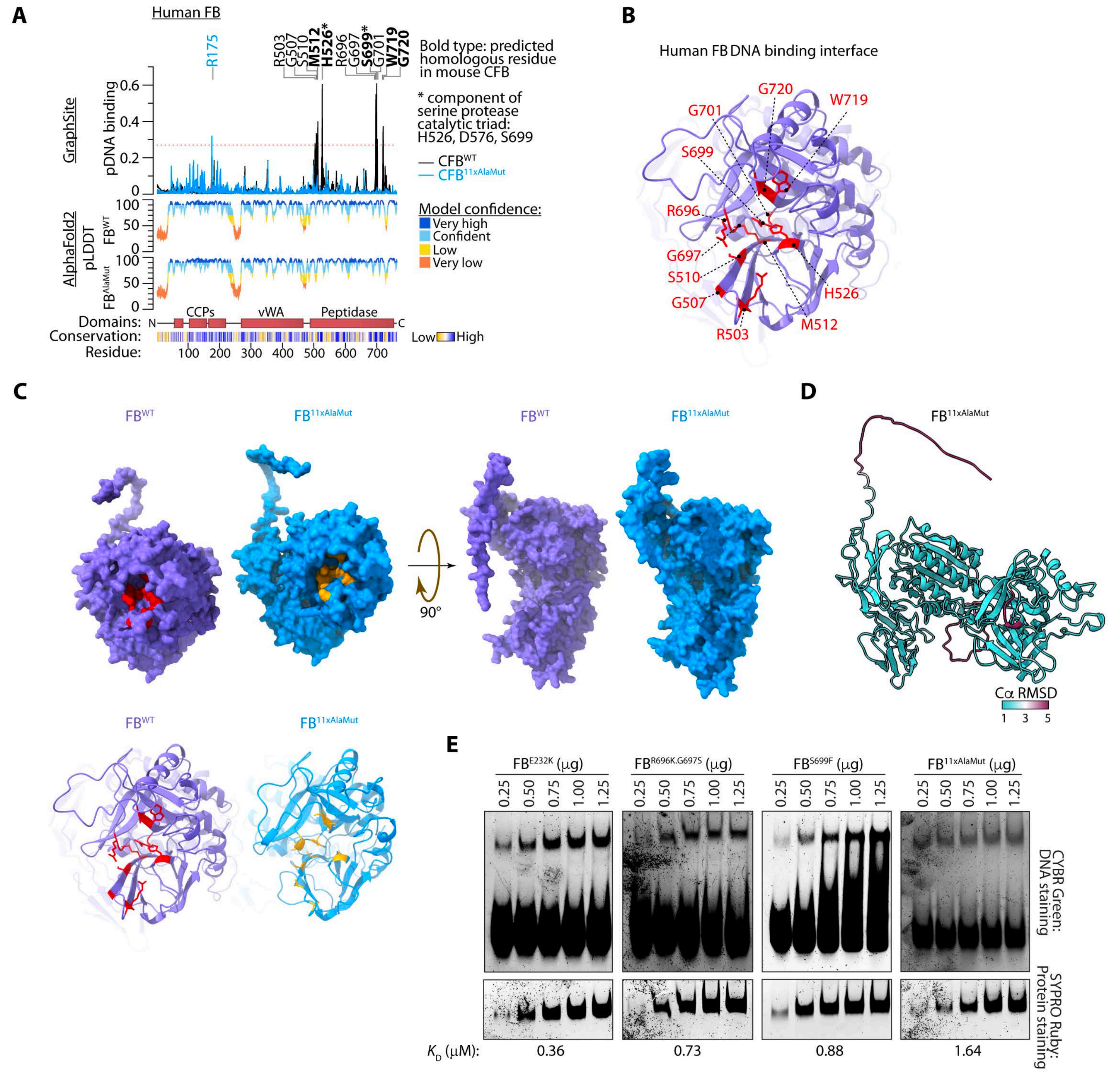

A
Human FB
R175
R503 G507 S510 M512 H526* R696 G697 S699* G701 W719 G720
Bold type: predicted homologous residue in mouse CFB
* component of serine protease catalytic triad: H526, D576, S699
— CFB^WT
— CFB^11xAlaMut
GraphSite
pDNA binding
AlphaFold2 pLDDT
FB^WT
FB^AlaMut
Model confidence:
Very high
Confident
Low
Very low
Domains:
CCPs
vWA
Peptidase
Conservation:
Low High
Residue:
B
Human FB DNA binding interface
G720
W719
G701
S699
R696
G697
S510
G507
R503
M512
H526
C
FB^WT
FB^11xAlaMut
90°
D
FB^11xAlaMut
Cα RMSD
E
FB^E232K (μg)
FB^R696K.G697S (μg)
FB^S699F (μg)
FB^11xAlaMut (μg)
0.25 0.50 0.75 1.00 1.25
CYBR Green: DNA staining
SYPRO Ruby: Protein staining
K_D (μM):
0.36
0.73
0.88
1.64

Figure S15

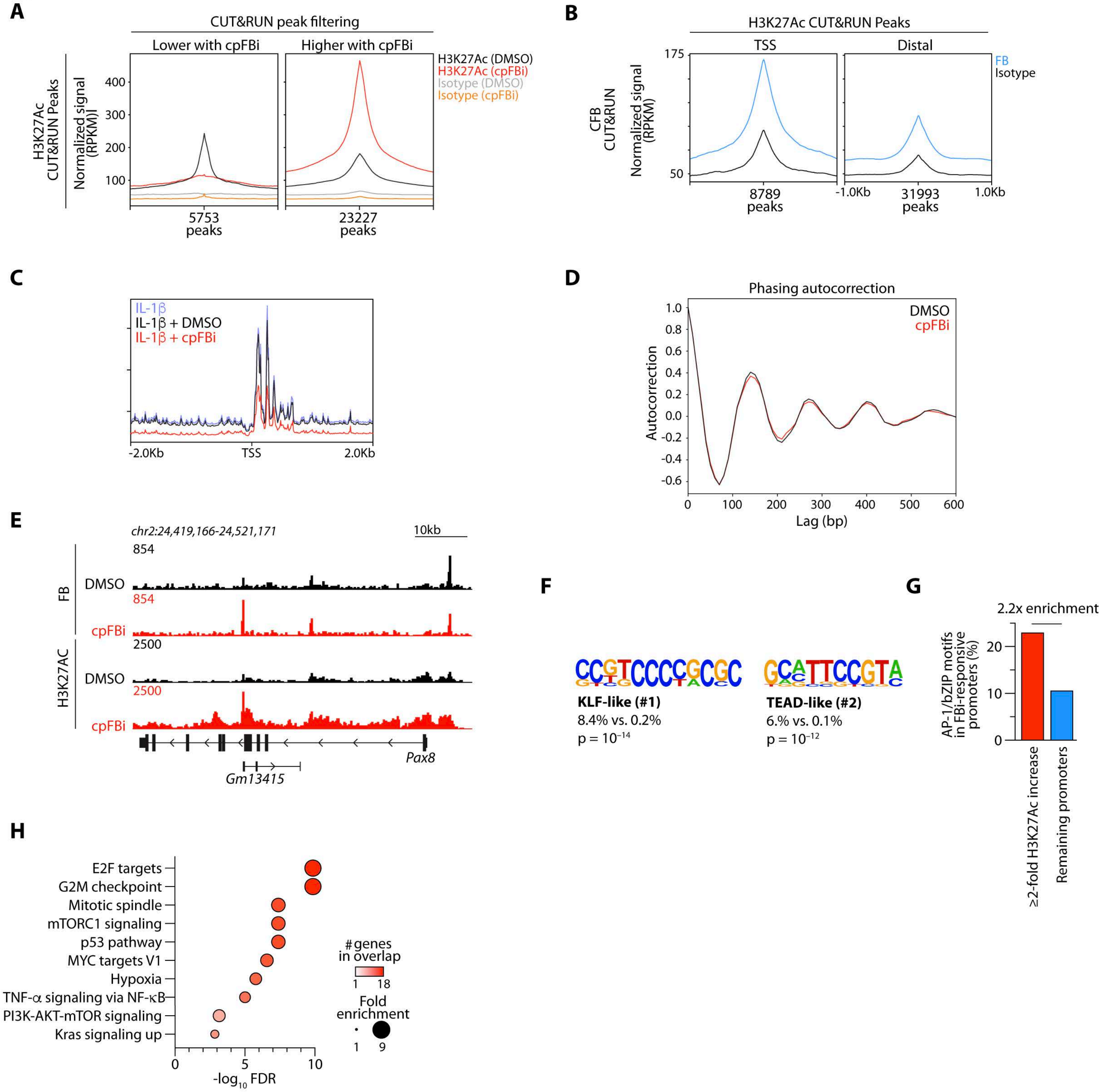

A
CUT&RUN peak filtering
Lower with cpFBi
Higher with cpFBi
H3K27Ac CUT&RUN Peaks
Normalized signal (RPKM)]
400
300
200
100
H3K27Ac (DMSO)
H3K27Ac (cpFBi)
Isotype (DMSO)
Isotype (cpFBi)
5753 peaks
23227 peaks
B
H3K27Ac CUT&RUN Peaks
TSS
Distal
CFB CUT&RUN
Normalized signal (RPKM)
175
50
FB
Isotype
8789 peaks
-1.0Kb
31993 peaks
1.0Kb
C
IL-1β
IL-1β + DMSO
IL-1β + cpFBi
-2.0Kb
TSS
2.0Kb
D
Phasing autocorrection
Autocorrection
1.0
0.8
0.6
0.4
0.2
0.0
-0.2
-0.4
-0.6
DMSO
cpFBi
0
100
200
300
400
500
600
Lag (bp)
E
chr2:24,419,166-24,521,171
10kb
FB
854
DMSO
854
cpFBi
H3K27AC
2500
DMSO
2500
cpFBi
Pax8
Gm13415
F
KLF-like (#1)
8.4% vs. 0.2%
p = 10^-14
TEAD-like (#2)
6.% vs. 0.1%
p = 10^-12
G
2.2x enrichment
AP-1/bZIP motifs in FBi-responsive promoters (%)
20
10
0
≥2-fold H3K27Ac increase
Remaining promoters
H
E2F targets
G2M checkpoint
Mitotic spindle
mTORC1 signaling
p53 pathway
MYC targets V1
Hypoxia
TNF-α signaling via NF-κB
PI3K-AKT-mTOR signaling
Kras signaling up
0
5
10
-log10 FDR
genes in overlap
1
18
Fold enrichment
1
9

Figure S16

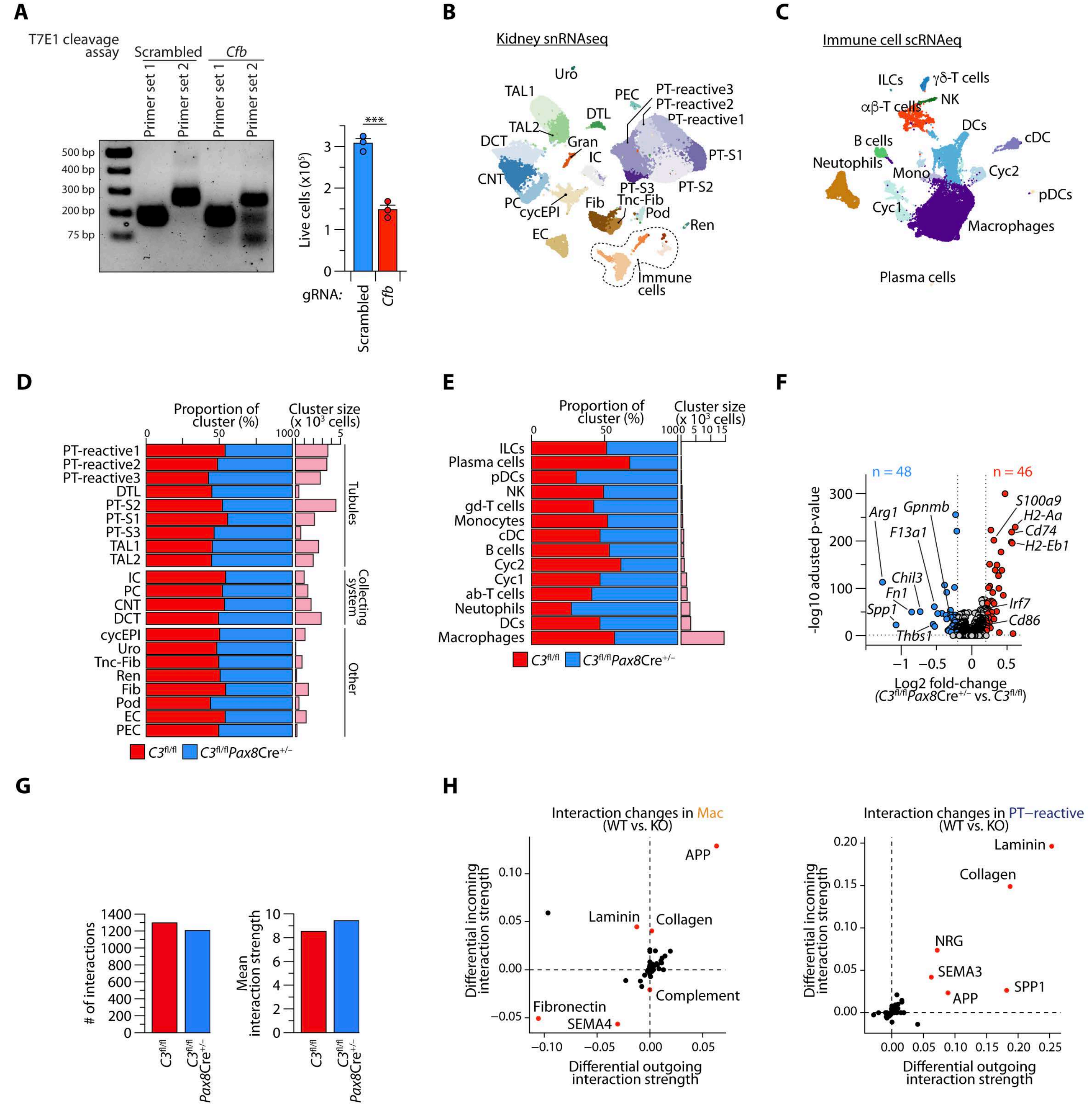
A
T7E1 cleavage assay
Scrambled
Cfb
Primer set 1
Primer set 2
Primer set 1
Primer set 2
500 bp
400 bp
300 bp
200 bp
75 bp
***
Live cells (x10^5)
gRNA:
Scrambled
Cfb
B
Kidney snRNAseq
Uro
TAL1
PEC
PT-reactive3
PT-reactive2
PT-reactive1
DTL
TAL2
Gran
DCT
IC
PT-S1
CNT
PT-S3
PT-S2
Tnc-Fib
PC
cycEPI
Fib
Pod
Ren
EC
Immune cells
C
Immune cell scRNAeq
ILCs
γδ-T cells
NK
αβ-T cells
DCs
B cells
cDC
Neutrophils
Mono
Cyc2
pDCs
Cyc1
Macrophages
Plasma cells
D
Proportion of cluster (%)
Cluster size (x 10^3 cells)
PT-reactive1
PT-reactive2
PT-reactive3
DTL
PT-S2
PT-S1
PT-S3
TAL1
TAL2
IC
PC
CNT
DCT
cycEPI
Uro
Tnc-Fib
Ren
Fib
Pod
EC
PEC
Tubules
Collecting system
Other
C3fl/fl
C3fl/fl Pax8Cre+/-
E
Proportion of cluster (%)
Cluster size (x 10^3 cells)
ILCs
Plasma cells
pDCs
NK
gd-T cells
Monocytes
cDC
B cells
Cyc2
Cyc1
ab-T cells
Neutrophils
DCs
Macrophages
C3fl/fl
C3fl/fl Pax8Cre+/-
F
n = 48
n = 46
-log10 adusted p-value
Arg1
Gpnmb
F13a1
Chil3
Fn1
Spp1
Thbs1
S100a9
H2-Aa
Cd74
H2-Eb1
Irf7
Cd86
Log2 fold-change
(C3fl/fl Pax8Cre+/- vs. C3fl/fl)
G
# of interactions
Mean interaction strength
C3fl/fl
C3fl/fl Pax8Cre+/-
H
Interaction changes in Mac
(WT vs. KO)
Differential incoming interaction strength
Differential outgoing interaction strength
APP
Laminin
Collagen
Complement
Fibronectin
SEMA4
Interaction changes in PT–reactive
(WT vs. KO)
Laminin
Collagen
NRG
SEMA3
APP
SPP1

Figure S17

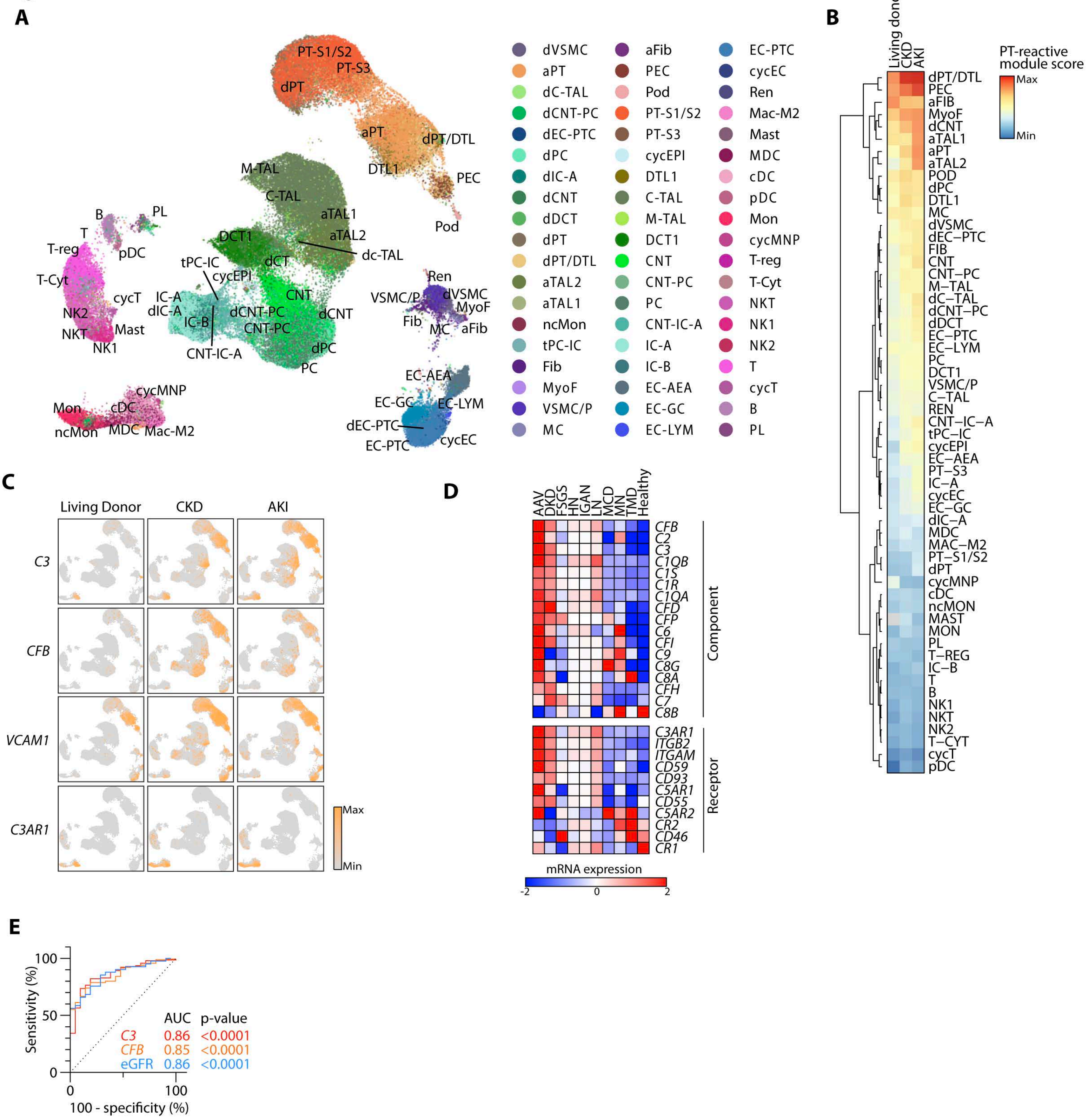

A
B
C
D
E
Living donor
CKD
AKI
PT-reactive module score
Max
Min
Living Donor
C3
CFB
VCAM1
C3AR1
AAV
DKD
FSGS
HN
IGAN
LN
MCD
MN
TMD
Healthy
Component
Receptor
mRNA expression
Sensitivity (%)
100 - specificity (%)
AUC p-value
C3 0.86 <0.0001
CFB 0.85 <0.0001
eGFR 0.86 <0.0001
